# Effects of Wildlife Feces on Soil Properties and Microbiota in the Recovery Processes of Damaged Natural Ecosystem

**DOI:** 10.1002/ece3.72423

**Published:** 2025-10-30

**Authors:** Yang Hong, Jiao Xiang, Han Wu, Tingfa Dong, Min Xu, Jinyan Huang, Zhenshan Guo, Yingchun Tan, Zhuo Tang, Lijie Chen, Zejun Zhang, Jindong Zhang

**Affiliations:** ^1^ Key Laboratory of Southwest China Wildlife Resources Conservation (Ministry of Education) China West Normal University Nanchong Sichuan China; ^2^ College of Wildlife and Protected Area Northeast Forestry University Harbin China; ^3^ College of Life Science China West Normal University Nanchong China; ^4^ Ministry of Education Key Laboratory for Transboundary Ecosecurity of Southwest China Kunming Yunnan China; ^5^ College of Environmental Science Sichuan Agricultural University Chengdu China; ^6^ Conservation and Research Centre for the Giant Panda/Key Laboratory of SFGA on Conservation Biology of Rare Animals in the Giant Panda National Park of China Dujiangyan China; ^7^ Administration Bureau of Wolong National Nature Reserve China; ^8^ Leshan Academy of Forestry Leshan China

**Keywords:** ecosystem restoration, microbiota, sambar ( *Rusa unicolor* ) feces, soil properties

## Abstract

Natural ecosystems are constantly damaged by natural or man‐made disasters. The restoration of damaged natural ecosystems is a systematic and intricate process. Wildlife feces can facilitate seed dispersal for plants and also influence soil properties and health. Soil restoration is the basis and prerequisite for the restoration of damaged ecosystems, and wildlife defecation is a key link for plant–animal–soil material turnover and nutrient cycling. Therefore, investigation of the effects of wildlife feces on soil properties and microbiota holds great significance for ecosystem restoration. To better understand the role of wildlife feces in the recovery of damaged natural ecosystems, we continuously monitored the areas destroyed by the 2008 earthquake for 15 years with a special focus on the feces of sambar ( *Rusa unicolor* ). Sambar shows the highest frequency of occurrence in the monitoring areas. We evaluated the effects of sambar feces on soil properties and microbial composition and function based on the feces‐to‐soil ratio obtained from field surveys (i.e., 0.14%). We found that the sambar feces changed multiple physical and chemical properties of soil; for example adding the 0.14% and 1.4% sambar feces to soil reduced the soil pH by 5.48% and 6.85%. PCoA results showed that adding sambar feces to soil significantly changed the composition of soil microbiota and reduced the diversity and abundance of soil bacteria in the short term. RDA exhibited that the pH, ${\mathrm{NO}}_{3}^{-}$‐N, C/N, and TN concentrations were the key physicochemical factors that significantly affect microbial diversity and composition. FAPROTAX predictions revealed that a high concentration of sambar feces (1.4%) would significantly increase the relative abundance of aerobic chemoheterotrophy and chemoheterotrophy (*p* < 0.05). Our study reveals the mechanisms by which wildlife feces affect soil restoration in damaged natural ecosystems. Because unpredictable disasters are ubiquitous worldwide, providing guidance for ecosystem restoration is of particular importance.

## Graphical abstract

Our findings provide a robust basis for future studies focused on revealing the mechanisms of animal–plant–soil interactions during the restoration of damaged ecosystems. Moreover, this study provides an example reference for guiding ecosystem restoration practices and makes up for the shortcomings of current research to promote the improvement of ecological restoration theory.

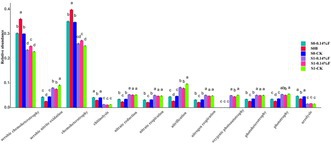

## 1 Introduction

Natural ecosystems are in constant cycles between being damaged by natural disasters (e.g., earthquake, landslide, debris flow, fires) or human disturbances (e.g., open‐cast mining, illegal reclamation as farmland) and restoration (Singh 1996; Dwivedi and Soni 2011; Wu et al. 2016; Ritonga et al. 2021; Cvetkovi et al. 2024). Studies have shown complex animal‐plant‐soil interactions during those processes (Macdonald et al. 2015). For example, after an earthquake, large areas of surface vegetation would be destroyed, leading to the formation of bare lands. Pioneer plants would then colonize the bare lands, initiating a new process of ecological succession, which would subsequently attract animals (e.g., ungulates) to participate in the succession (Guo et al. 2022). The presence of animals would facilitate seed dispersal (Bullock et al. 2011; Baltzinger et al. 2019), while animal feces would affect the physicochemical properties of soil as well as the composition and function of the soil microbiota (Lazcano et al. 2021). Soil is a crucial component of terrestrial ecosystems which shows complex structures and functions. It is a fundamental requirement for the survival of terrestrial plants, which form important habitats for many animals and provide necessary resources for human survival (Xiong 1983; Al‐Kaisi et al. 2017). Moreover, soil is the most active place for material exchange and energy flow between organisms and their environments (Xiong 1983; Bastida et al. 2021). The ecological functions of soil are related to the overall health of the entire terrestrial ecosystem. The functional restoration of soil ecosystems is a prerequisite for the restoration of any damaged ecosystem. Therefore, soil restoration is of top priority in the repair processes of damaged ecosystems.

The feces of animals are considered important sources of nutrients in the damaged terrestrial systems (Haynes and Williams 1992; White et al. 2001). Animal feces can not only increase soil nutrients, but can also accelerate the rate of nutrient cycling (Tayyab et al. 2018; Bhunia et al. 2021). Studies have shown that nutrients tend to be released more rapidly from animal feces compared to plant litters (Ruess and Mcnaughton 1987; Saarijarvi and Virkajarvi 2009). In addition, animal feces can also impact the soil properties and soil microbiota (Lazcano et al. 2021). Animal feces affect soil properties and microbes can be divided into two categories based on the characteristics of animals, that is livestock and wildlife. There are abundant studies on how livestock feces affect soil and microbes. For example, Sun et al. showed that the addition of feces from domestic pig (*Sus scrofa f. domestica*) or cattle (*Bos taurus domesticus*) to long‐term farming soils significantly restored bacterial diversity to the same level as non‐farming status (Sun et al. 2015). As another example, Zhang indicated that cattle manure decomposition has an impact on soil physicochemical properties, microbial biomass and enzyme activity (Zhang 2019). However, the impact of wildlife feces on soil properties and soil microbiota remains significantly underexplored currently. The small populations of wildlife in some studied areas and the difficulties associated with collecting enough experimental materials have impeded detailed studies in this field.

Throughout the long‐term monitoring period, we found that a large number of plant‐eating animals including sambar deer ( *Rusa unicolor* ), Chinese gorals ( *Naemorhedus griseus* ), Chinese serows ( *Capricornis milneedwardsii* ), and tufted deer ( *Elaphodus cephalophus* ) had fed in the earthquake‐damaged areas (Zhang, Wang, et al. 2011; Xiang et al. 2022). These herbivores are known to play crucial roles in the restoration of vegetation in the earthquake‐damaged areas. Herbivores can not only affect the structure of vegetation by eating and trampling on plants (Bullock et al. 2011; Albert et al. 2015), but can also spread seeds through feces (Mélanie et al. 2015). Meanwhile, their feces and urine can also affect the soil where plants grow. When the feces are deposited onto the soil surface, they become an essential source of material and energy for the soil microbiota and provide an important way for the nutrients to be returned to plants (Zhang 2019). The wild ungulates that settle in the seismic disaster areas produce a substantial amount of feces and therefore provide valuable opportunities for investigation on how wild animals influence soil properties and health. During the monitoring period, we found that sambars utilized the earthquake disaster areas most frequently among large ungulates. We also quantified the ratio of sambar feces to soil. On the basis of this ratio, we designed controlled experiments to examine the effects of sambar feces on soil properties and soil microbiota.

This study answers the following scientific questions: (1) How do soil physicochemical properties change in response to sambar feces application? (2) How does the addition of sambar feces affect soil microbial composition? (3) Does the addition of sambar feces affect the correlations between soil microorganisms and environmental factors? and (4) Is it possible to predict the functional variations of soil microorganisms after the addition of sambar feces? The controlled experiments in this study are helpful for elucidating the critical roles that wildlife play in the restoration processes of damaged forest ecosystems. They also enhance our understanding of the complex coupling relationships among animals, plants and soil, thereby enriching the theoretical framework of restoration ecology. Additionally, they provide valuable scientific references for restoration efforts.

## 2 Materials and Methods

### 2.1 Study Areas

**FIGURE 1:**
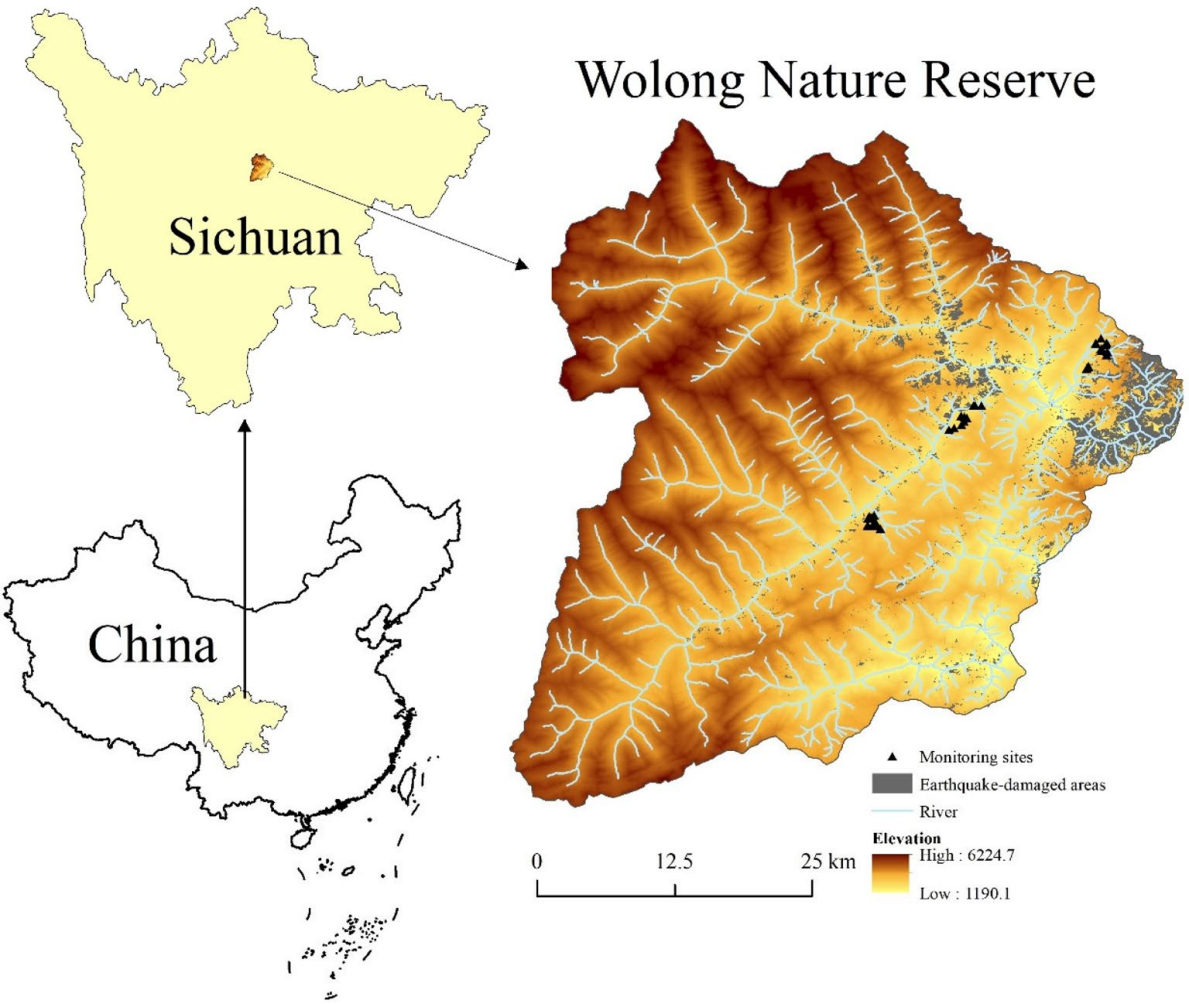
Location of study area and distribution of monitoring earthquake‐affected sites.

Our monitoring of earthquake disaster areas, collection of sambar feces and soil and other control experiments were all performed within the Wolong Nature Reserve (102°52′–103°24′ E, 30°45′–31°25′ N), Sichuan, China (Figure 1) (Liu et al. 2001). The reserve covers 2000 km^2^, with an elevation range of 1190.1–6224.7 m, and there are abundant animal and plant resources. It provides a home for many endangered animals, including giant panda ( *Ailuropoda melanoleuca* ), snow leopard (*Panthera uncia*) and Sichuan snub‐nosed monkey ( *Rhinopithecus roxellana* ) (Hou et al. 2018). The vegetation communities in Wolong can be categorized into six distinct types according to altitude: evergreen broad‐leaved forest, evergreen and deciduous broad‐leaved mixed forest, coniferous and broad‐leaved mixed forest, cold‐temperate coniferous forest, cold‐resistant shrubland, alpine meadow, and sparse vegetation on alpine talus slopes. The vegetation cover of Wolong exceeds 98%, of which 57.6% is forested. The reserve encompasses a wide variety of forest types, among which the alpine forest ecosystem is considered the most representative. The forests are predominantly original and naturally developed, although a small proportion consists of artificial plantations, such as restoration forests established in landslide‐affected areas following the Wenchuan earthquake. Wolong is located on the Longmen Mountain Fault, which is prone to frequent earthquakes (Ouyang et al. 2008; Zhang et al. 2014). During the 2008 Wenchuan earthquake, Wolong was among the most severely affected regions, with numerous natural ecosystems within the reserve suffering significant damage. To assess the post‐earthquake recovery of Wolong, we selected 40 earthquake‐affected areas (Table S1) from all damaged areas previously encountered during initial field surveys conducted in 2008 (*n* = 107) (Figure 1) (Ouyang et al. 2008; Zhang et al. 2014) and conducted a 15‐year (up to 2023) monitoring program with a special focus on vegetation recovery and animal utilization (Zhang et al. 2014, 2023; Xiang et al. 2022). Annually, we recorded data on vegetation recovery and wildlife utilization. In our 2022 study, we observed that at least 28 species of wildlife currently utilize the earthquake disaster areas. including wild boars ( *Sus scrofa* ), sambars, tufted deer ( *Elaphodus cephalophus* ), Chinese serows ( *Capricornis milneedwardsii* ), and Chinese gorals ( *Naemorhedus griseus* ) (Guo et al. 2022). During the process of vegetation restoration, the pioneer dominant plant species mainly include *Rubus thibetanus*, *Buddleja davidii* , *Salix rehderiana*, and *Betula albosinensis*. Vegetation cover initially increases and subsequently stabilizes over time (Guo 2022; Xiang et al. 2022). Notably, sambars utilize the disaster areas with consistently high frequencies (Xiang et al. 2022). Therefore, we selected sambar feces as an example in this study. The Wolong Nature Reserve shows a typical subtropical inland mountain climate, with a mean air temperature around 8.4°C, and an average annual precipitation of 861.8 mm, mainly in summer. The soil in the research areas belongs to the mountain brown soil (Zhang et al. 2024).

There are two key drivers that led us to select Wolong as the research site. (1) Wolong is a well‐recognized biodiversity hotspot that experienced severe damage from the 2008 M‐earthquake, triggering extensive secondary succession. This unique setting provides an ideal natural laboratory to investigate the interrelationships among vegetation succession, animal utilization and soil microorganisms. (2) The wild ungulates, especially the sambars, prefer to feed on pioneer plants in earthquake‐damaged areas (such as *Rubus idaeus* and *Buddleja davidii* ), which provides a real ratio of soil to wildlife feces. This study was performed based on real monitoring data; there was clear theoretical significance and practical value for ecological restoration.

### 2.2 Preliminary Experiment

The utilization rate and frequency of sambar visits to earthquake‐damaged areas were comparatively high (Xiang et al. 2022). Therefore, the sambar feces were selected for this study. To determine the ratio of sambar feces to soil on the earthquake‐damaged areas in the field environment, a preliminary experiment was performed. In order to clear the ratio of sambar feces to soil under natural conditions. sampling plots were established for the collection of feces with a pile of sambar feces as the center point. In June 2021, we randomly selected 10 monitoring sites from a total of 40 (average size was 3578 ± 541 m^2^, range 15–180,000m^2^) (Zhang et al. 2014). Within each selected monitoring site, one 1 m × 1 m feces collection plot was established. Inside each feces collection plot, 10 soil sampling subplots (10 cm × 10 cm × 20 cm) were set up. The sampling personnel wore disposable gloves and carefully collected all sambar feces in the feces collection plots into disposable plastic‐sealed bags. The temperature of the sampling site was used as an indicator and was kept at a constant temperature to be taken back to the laboratory. The samples were subsequently dried in a soil drying oven at 105°C and then weighed using an electronic balance to determine their dry mass (O'Kelly and Sivakumar 2014), from which an average weight of sambar feces *m*_1_ was obtained. At the same time, the researcher wore new disposable gloves to remove the surface litter layer, and soil samples were collected from 0 to 20 cm below the surface using a specialized soil sampler (Dandwate 2020; She et al. 2023). Subsequent treatment of soil samples was done in the same manner as for sambar feces. Then, the average weight *m*_2_ of the soil was obtained. Drying and weighing experiments of sambar feces and soil were conducted in the Key Laboratory of Southwest China Wildlife Resources Conservation (Ministry of Education), China West Normal University, Nanchong, Sichuan Province, China. The ratio of feces to soil S under field conditions was calculated using the following equation:

$$S=\left(m_{1}/100\right)/m_{2}$$

The results from the preliminary field experiment (total 100 soil samples) showed that *S* = 0.14 (i.e., each 100 g of soil contained 0.14 g of sambar feces).

### 2.3 Soil and Sambar Feces for Pot Incubation Experiment

The sampling method for sambar feces used in the pot cultivation experiment was consistent with that employed in the preliminary experiment. The collected sambar feces were physically crushed using a soil grinding instrument. Experimental personnel wore disposable gloves and manually homogenized the samples completely. Using an electronic balance, the feces material was divided into two equal portions based on weight. One portion was subjected to enhanced ventilation under the sampling temperature to facilitate natural air drying, then sieved through a 0.15 mm mesh to remove indigestible plant residues, used for the analysis of soil physicochemical properties. Another portion was stored without additional ventilation under the same temperature conditions for use in the pot incubation experiments.

For the collection of soil used in the pot incubation experiments, we randomly selected one monitoring site (103°18′ E, 31°03′ N) from a total of 40 and established five 1 m × 1 m soil sampling plots in the east, south, west, north, and central areas of the selected site to obtain sufficient soil material. The rationale for not collecting soil from all monitoring sites was as follows: if soil were collected from each of the earthquake‐damaged monitoring sites and subsequently mixed for use in the pot cultivation experiment, variations in soil quality, altitude, and other environmental factors across the sites would lead to differences in soil properties and microbial composition. Mixing such heterogeneous soils would result in an artificial soil matrix that does not naturally occur in Wolong. Given this consideration, we chose to collect soil from only one representative monitoring site for the pot cultivation experiment. Soil sample preparation followed the same procedure as that for sambar feces, with the sole exception that soil intended for the pot incubation experiments was not subjected to the grinding process.

The physiochemical properties of the soil and sambar feces were analyzed according to the procedures described in “Agricultural Chemistry Analysis” (Bao 2000). The pH of the samples was measured using a pH electrode (Leici, Shanghai, China) with a soil/feces: water ratio of 1:2.5. The water content was determined by the mass loss from approximately 10 g of soil or feces after incubation at 105°C for at least 8 h (Bao 2000). The total carbon (TC) and total nitrogen (TN) were determined by dry combustion analysis on an elemental analyzer (Vario TOC, Germany). Total phosphorus (TP) was dissolved by H_2_SO_4_‐HClO_4_ and then determined with a spectrophotometer (TU‐1810, Beijing, China). The ${\mathrm{NO}}_{3}^{-}$‐N and ${\mathrm{NH}}_{4}^{+}$‐N concentrations were determined by continuous flow (DeChem‐Tech., GmbH, Germany) after extraction with 2 M KCl. The original physiochemical properties of soil and sambar feces are shown in Table 1.

**TABLE 1:** Original physical and chemical properties of the tested soil sample and sambar feces.

Items	pH	Water content (%)	TC (%)	TN (%)	TP (%)	${\mathrm{NH}}_{4}^{-}$‐N (mg/kg)	${\mathrm{NO}}_{3}^{-}$ ^_^N (mg/kg)
Soil	7.3	11.6	3.295	0.106	1.236	3.946	2.299
Sambar feces	8.1	268.1	40.439	2.541	0.258	12.36	249.83

### 2.4 Pot Incubation Experiment

To investigate the effects of different amounts of sambar feces on the physiochemical properties of soil and the microorganisms that occupied the soil sample, incubation experiments were performed with the addition of sambar feces at ratios of 0.14% (i.e., the ratio of sambar feces to soil under natural conditions) and 1.4% (i.e., 10 times the proportion of sambar feces to soil under natural conditions was used to study the effects of excessively high sambar feces concentration on soil properties and microorganisms) to soil, defined as 0.14%F and 1.4%F in this paper, respectively. Soil without feces was used as a control (CK). The three treatments (0.14%F, 1.4%F, and CK) were each replicated 15 times. The soil and sambar feces were mixed and placed into plastic pots (upper diameter: 20 cm; lower diameter: 15 cm; height: 20 cm). Each pot was loaded with 3 kg of soil. The pots were placed in the open field in Wolong. The duration of the pot experiment was 30 days (i.e., June 2021–July 2021), and soil samples were collected after day 0 (S0) and day 30 (S1) after the application of sambar feces. Soil samples were divided into two portions. One portion was dried under room temperature to analyze soil characteristics, and another part was stored at −80°C for molecular analysis of the soil microbiota (Bao 2000; Ren et al. 2016; Qi et al. 2021).

### 2.5 DNA Extraction and Illumina MiSeq Sequencing

Soil microbial DNA was extracted using cetyltrimethylammonium bromide (CTAB). Staining with ethidium bromide (EB), DNA concentration, and purity were determined with 1% agarose gels. DNA was diluted to 1 ng/μL using sterile water. 16S rRNA genes of distinct regions (16S V3‐V4) were amplified using a specific primer pair (341F (5′‐CCTAYGGGRBGCASCAG‐3′) and 806R (5′‐GGACTACNNGGGTATCTAAT‐3′)) with barcodes. All PCR reactions were performed with 15 μL of Phusion High‐Fidelity PCR Master Mix (New England Biolabs), 2 μM of forward and reverse primers, and about 10 ng of template DNA. Thermal cycling consisted of initial denaturation at 98°C for 1 min, followed by 30 cycles of denaturation at 98°C for 10 s, annealing at 50°C for 30 s, and elongation at 72°C for 30 s. A final step of elongation was performed at 72°C for 5 min after the cycles. The same volume of 1xTAE buffer was mixed with PCR products and the products were detected by electrophoresis on a 2% agarose gel. PCR products were mixed in equal ratios using a vortex oscillator. Then, the mixture of PCR products was purified with the Qiagen Gel Extraction Kit (Qiagen, Germany). Sequencing libraries were generated using the TruSeq DNA PCR‐Free Sample Preparation Kit (Illumina, USA) following manufacturer's recommendations and index codes were added. The library quality was assessed on the Qubit@ 2.0 Fluorometer (Thermo Scientific). After confirmation of the library quality, the library was sequenced on an Illumina NovaSeq platform and 250 bp paired‐end reads were generated.

### 2.6 Bioinformatics Analysis

The analysis was conducted following the “Atacama soil microbiome tutorial” of Qiime2docs together with customized program scripts (https://docs.qiime2.org/2019.1/). Briefly, raw FASTQ files were transformed into a format which could be recognized by the QIIME2 system using qiime tools. Demultiplexed sequences from each sample were quality filtered and trimmed, de‐noised, and merged. The chimeric sequences were identified and removed using the QIIME2 dada2 plugin to obtain the feature table of amplicon sequence variant (ASV) (Callahan et al. 2016). The QIIME2 feature‐classifier plugin was then used to align ASV sequences to a pre‐trained GREENGENES 13_8 99% database (trimmed to the V3V4 region bound by the 338F/806R primer pair) to generate the taxonomy table (Bokulich et al. 2018). All contaminated mitochondrial and chloroplast sequences were filtered using the QIIME2 feature‐table plugin.

### 2.7 Data Analysis

All the data are presented as mean ± standard error. A one‐way analysis of variance (ANOVA) followed by Duncan's multiple range test (*p* < 0.05) was performed to compare the difference in the physiochemical properties among the soil samples, the α‐diversity indices and relative abundance of soil microbial communities between treatments using IBM SPSS v25.0 (IMB Corp., Armonk, USA). OTUs (Operational Taxonomic Units), Chao1, and Shannon indices were used to assess the microbial alpha diversity. Principal Coordinate Analysis (PCoA) based on Bray–Curtis distance between samples was calculated as the β diversity of microbial communities (compositional dissimilarity between fields) using the Phyloseq package in R. Nonmetric multidimensional scaling (NMDS) analysis was also performed using the R Phyloseq package. The correlations between the soil parameters and the soil samples were illustrated by the redundancy analysis (RDA), which was performed using CANOCO 5.0. Network analysis in R using the Igraph package was performed to study the relationship between soil parameters and the relative abundances of specific bacterial taxa. After obtaining the identification and abundance information of the OTUs, functional prediction was achieved through the FAPROTAX module (http://www.ehbio.com/ImageGP/). Heat map analysis in R using the Pheatmap package was performed to explore the relationship between specific functional groups, soil parameters and soil microorganisms.

## 3 Results

### 3.1 Impact of Sambar Feces on the Physicochemical Properties of Soil

The soil pH in the 0.14%F and 1.4%F groups was significantly lower than that of the CK groups across all samples. The TC of the 1.4%F samples was higher than that in the 0.14%F and CK samples, and the ${\mathrm{NO}}_{3}^{-}$ ^_^N of 0.14%F and 1.4%F samples was significantly lower compared to CK samples in the S0. In the S1 stage, the TC of CK samples was significantly lower than that of 0.14%F and 1.4%F, and the TN concentration of the 0.14%F and 1.4%F samples significantly decreased compared to CK. The C/N ratios in 0.14%F and 1.4%F samples remained constant in the S0 stage and increased significantly in the S1 stage. (Table 2).

**TABLE 2:** Physical and chemical properties of the soils at different time points.

Stages	Treatments	pH	Water content (%)	TC (%)	TN (%)	TP (%)	${\mathrm{NH}}_{4}^{-}$ ^_^N (mg/kg)	${\mathrm{NO}}_{3}^{-}$ ^_^N (mg/kg)	C/N
S0	CK	7.3 ± 0.1c	11.6 ± 1.5b	3.29 ± 0.09ab	0.11 ± 0.01c	1.24 ± 0.25a	3.95 ± 1.35b	2.29 ± 0.31a	31.37 ± 3.34a
	0.14%F	6.9 ± 0.1d	12.1 ± 1.3b	3.22 ± 0.08ab	0.10 ± 0.08c	1.22 ± 0.32a	3.61 ± 1.24b	1.96 ± 0.16b	31.53 ± 2.94a
	1.4%F	6.8 ± 0.1d	12.9 ± 1.5b	3.38 ± 0.09a	0.10 ± 0.01c	1.21 ± 0.20a	3.51 ± 0.76b	1.76 ± 0.41b	32.60 ± 3.92a
S1	CK	8.0 ± 0.2a	17.5 ± 1.7a	2.77 ± 0.32c	0.14 ± 0.01a	1.18 ± 0.55a	6.03 ± 1.34a	0.17 ± 0.09c	20.01 ± 2.31c
	0.14%F	7.9 ± 0.1b	17.8 ± 1.3a	3.20 ± 0.63ab	0.12 ± 0.08b	1.39 ± 0.35a	5.45 ± 0.78a	0.18 ± 0.06c	24.96 ± 4.98b
	1.4%F	7.9 ± 0.1b	17.6 ± 3.3a	3.06 ± 0.08b	0.12 ± 0.01b	1.36 ± 0.16a	5.60 ± 1.24a	0.20 ± 0.09c	23.87 ± 2.11b

*Note:* S0: samples at day 0 after sambar feces application. S1: samples at day 30 after sambar feces application. CK, 0.14%F and 1.4%F represent sambar feces addition at feces to soil ratios of 0%, 0.14% and 1.4%, respectively. Different letters (i.e., a, b, c and d) in the same column indicate significant differences at *p* < 0.05.

### 3.2 Impact of Sambar Feces on Soil Microbial Diversity and Composition

In the S0 stage, the Chao1 index, number of OTUs and Shannon index in 1.4%F were significantly lower than those in CK. The number of OTUs and the Chao1 index in 1.4%F were significantly lower than those in CK in the S1 stage (Table 3).

**TABLE 3:** Variations in the functional diversity indices of the soil microbiota with different treatments.

Periods	Treatments	OTUs	Chao1	Shannon
S0	CK	2212 ± 150c	2245 ± 155c	10.1 ± 0.1b
	0.14%F	2150 ± 253 cd	2016 ± 256d	10.1 ± 0.2b
	1.4%F	1988 ± 208d	2180 ± 208d	9.8 ± 0.3c
S1	CK	2849 ± 131a	2888 ± 133a	10.7 ± 0.0a
	0.14%F	2682 ± 249ab	2714 ± 257b	10.6 ± 0.2a
	1.4%F	2656 ± 346b	2693 ± 356b	10.6 ± 0.2a

*Note:* The meanings represented by S0, S1, and various additional letters (i.e., a, b, c, d) are in accordance with those in Table 2.

**FIGURE 2:**
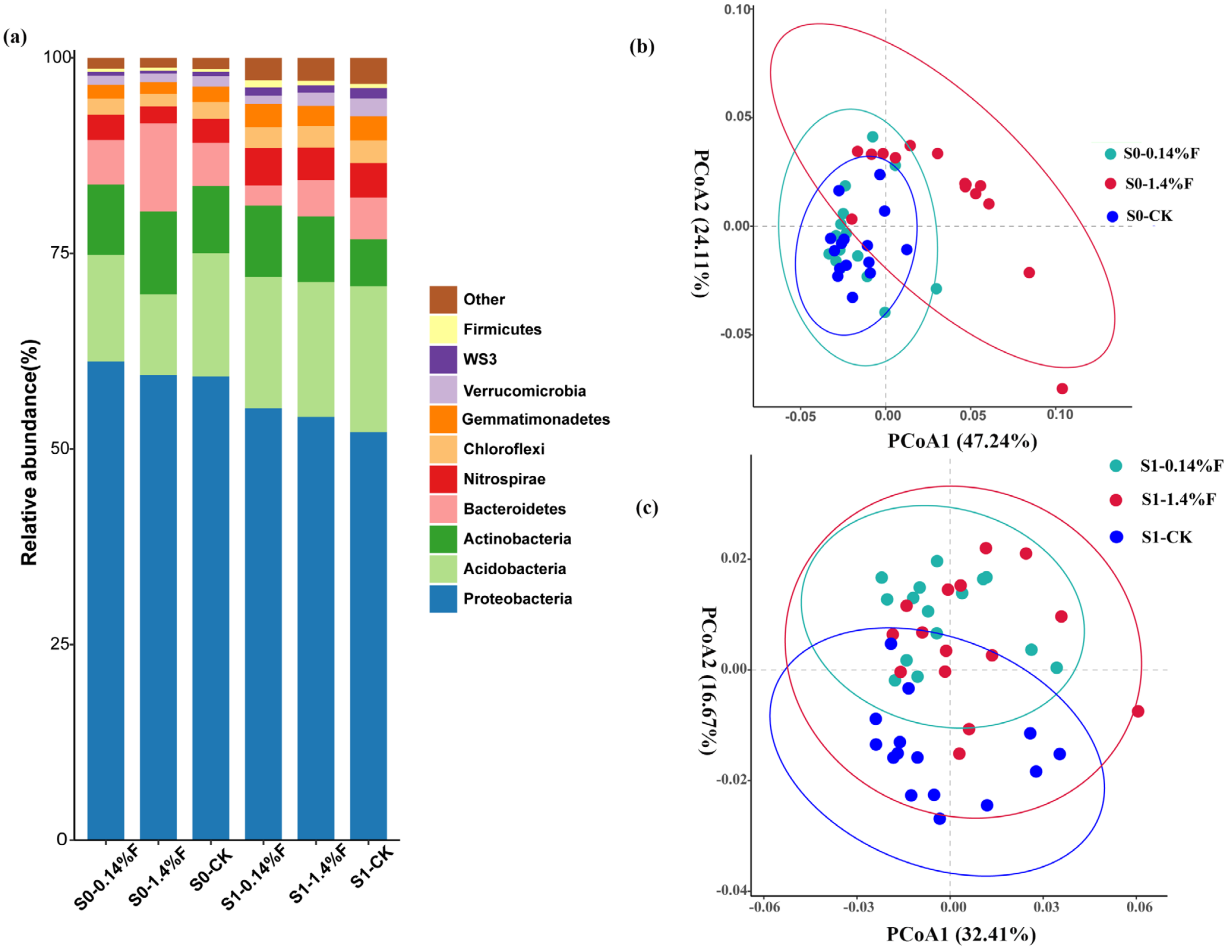
(a) Relative distribution of different microbial groups with different treatments at the level of phylum. (b, c) Principal coordinate analysis (PCoA) of soil bacteria with different treatments in S0 and S1periods. S0: Samples at day 0 after sambar feces application. S1: Samples at day 30 after sambar feces application. The CK, 0.14%F and 1.4%F represent sambar feces addition at feces to soil ratios of 0%, 0.14% and 1.4%, respectively.

The main purpose of Beta diversity analysis was to examine the similarity of community structure among different samples. The results showed that there were significant differences in microbial community structures of soils among different treatments at each stage. The microbial communities of 1.4%F were separated from those of 0.14%F and CK in the S0 stage (Figure 2b). In the S1 stage, soil bacterial communities of the 0.14%F and 1.4%F samples clustered together compared with CK. (Figure 2c).

At the phylum level, the application of 0.14% and 1.4% sambar feces both significantly affected the abundance of different bacterial groups. The soil in S0 period was dominated by *Proteobacteria* (59.26%–61.19%), *Acidobacteria* (10.35%–15.75%), *Actinobacteria* (8.62%–10.57%), *Bacteroidetes* (5.51%–11.26%), *Nitrospirae* (2.16%–3.23%), and *Chloroflexi* (2.06%–2.15%), which together account for 94.36%–95.38% of the bacterial sequences. The taxonomic predominance of soil microbial at day 30 changed slightly. The abundance of *Proteobacteria* (52.15%–55.21%), *Actinobacteria* (6.00%–9.12%), and *Bacteroidetes* (2.57%–5.33%) decreased, and the abundance of *Acidobacteria* (16.79%–18.69%), *Nitrospirae* (4.44%–4.77%), and *Chloroflexi* (2.67%–2.89%) increased. Taken together, those bacterial groups account for 89.45%–91.29% of the bacterial sequences (Figure 2a).

In the S0, compared with CK, application of sambar feces in the 0.14%F and 1.4%F samples increased the relative abundance of *Proteobacteria*, *Actinobacteria*, *Bacteroidetes*, and *Firmicutes*, but decreased the relative abundance of *Acidobacteria, Chloroflexi*, *Gemmatimonadetes*, *Verrucomicrobia*, and *WS3*. In the S1 stage, a comparison between the relative abundance of soil microbes in the 0.14%F and 1.4%F samples and that of the CK samples shows that the relative abundance of *Proteobacteria*, *Actinobacteria*, and *Firmicutes* increased, but the relative abundance of *Acidobacteria*, *Bacteroidetes*, *Chloroflexi*, *Verrucomicrobia*, and *WS3* decreased.

### 3.3 Relationship Between Soil Microbiota and Physicochemical Properties

**FIGURE 3:**
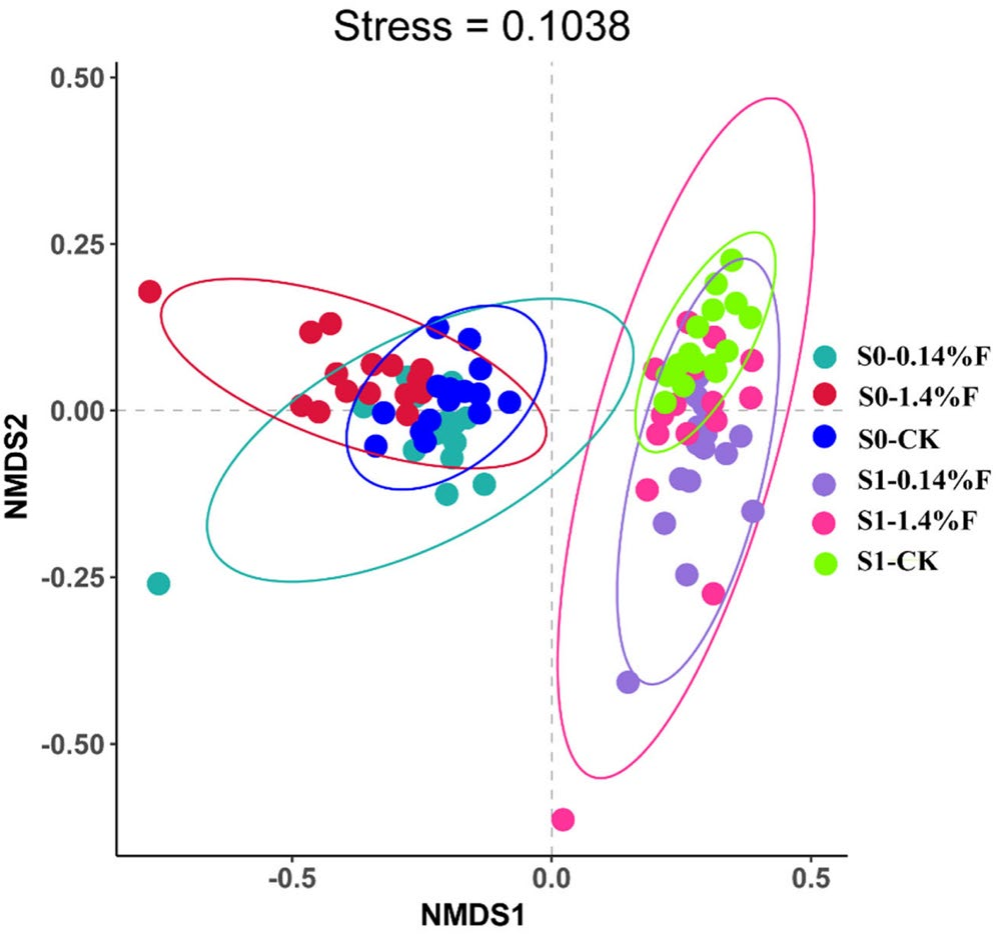
Nonmetric multidimensional scaling (NMDS) analysis of soil bacteria from different treatments at different times.

A separation in soil microbiota compositions after sambar feces application at day 0 and day 30 was clearly observed (Figure 3), which indicated that sambar feces application results in a large variation in the bacterial community structure.

**FIGURE 4:**
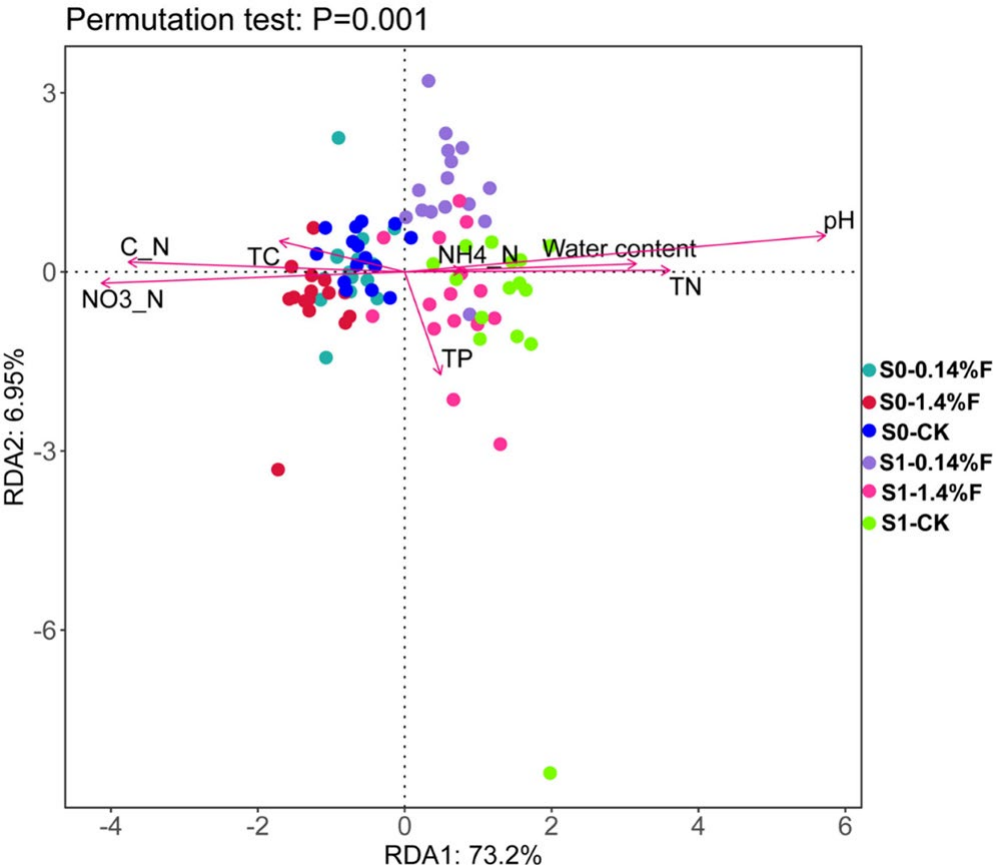
Redundancy analysis (RDA) of the correlations between the soil parameters and soil microbiota.

Redundancy analysis (RDA) was performed on the correlation between changes in the soil microbiota and soil physicochemical properties with different treatments. The first two RDA axes explained 73.2% and 6.95% of the total variation in the data. RDA confirmed that pH (*F* = 71.9, *p* = 0.002), ${\mathrm{NO}}_{3}^{-}$_N (*F* = 37.9, *p* = 0.002), C/N (*F* = 36.3, *p* = 0.002), and TN (*F* = 34.3, *p* = 0.002) were the most important contributors to the variation in bacterial communities and accounted for 89.3%, 59.8%, 58.0%, and 55.7% of the total variation, respectively (Figure 4).

**FIGURE 5:**
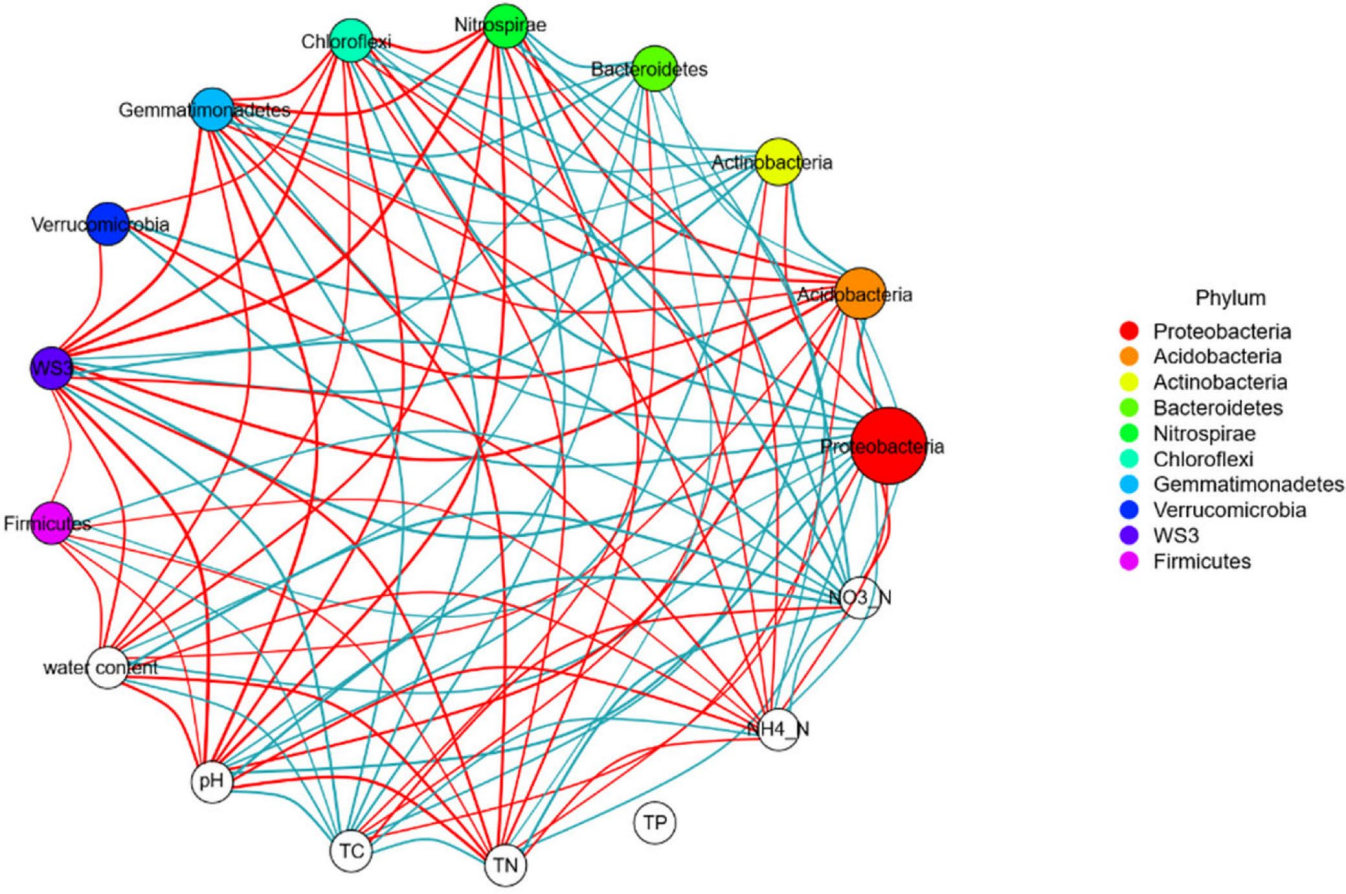
Network analysis between dominant bacteria and soil physical and chemical properties. Each solid circle represents a species, and the sizes and colors represent its relative abundance and different phyla, respectively. Open circles represent soil physicochemical factors. The lines between circles represent significant correlations between the two species or soil properties (*p* < 0.05). Red lines represent positive correlation and blue lines represent negative correlation. The thickness of the line represents different absolute values of the correlation coefficient.

Network analysis is beneficial to explain the correlation between soil microorganisms and soil physicochemical properties. On the basis of the network, the pH, TN and NH_4_^+^_N had a significant negative correlation with *Proteobacteria*, *Bacteroidetes*, and *Actinobacteria*, but had a significant positive correlation with *Acidobacteria* and *Nitrospirae*. The TC and ${\mathrm{NO}}_{3}^{-}$_N had a significant positive correlation with *Proteobacteria*, *Bacteroidetes*, and *Actinobacteria*, but had a significant negative correlation with *Acidobacteria* and *Nitrospirae* (Figure 5).

### 3.4 Functional Prediction of Soil Bacteria by FAPROTAX

We inquired about potential functional groups involved in the formation of environmental conditions in soil. From the classifying annotation results of the 16S rDNA sequences, a total of 74 functional groups were identified using FAPROTAX. The core functional groups (average relative abundance > 1%) of the soil microbiota in the S0 time point are chemoheterotrophy (28.72%), aerobic chemoheterotrophy (25.30%), nitrification (2.97%), aerobic nitrite oxidation (2.86%), phototrophy (2.50%), nitrate reduction (2.38%), photoheterotrophy (2.48%), nitrogen respiration (2.20%), ureolysis (2.89%), and nitrate respiration (2.19%). The dominant functional groups changed slightly at day 30. The average relative abundance of chemoheterotrophy (18.42%), aerobic chemoheterotrophy (16.72%), and ureolysis (1.12%) decreased, while the average relative abundance of nitrification (5.87%), aerobic nitrite oxidation (5.64%), phototrophy (3.78%), nitrate reduction (3.70%), photoheterotrophy (3.55%), nitrogen respiration (3.46%), and nitrate respiration (3.44%) increased.

**FIGURE 6:**
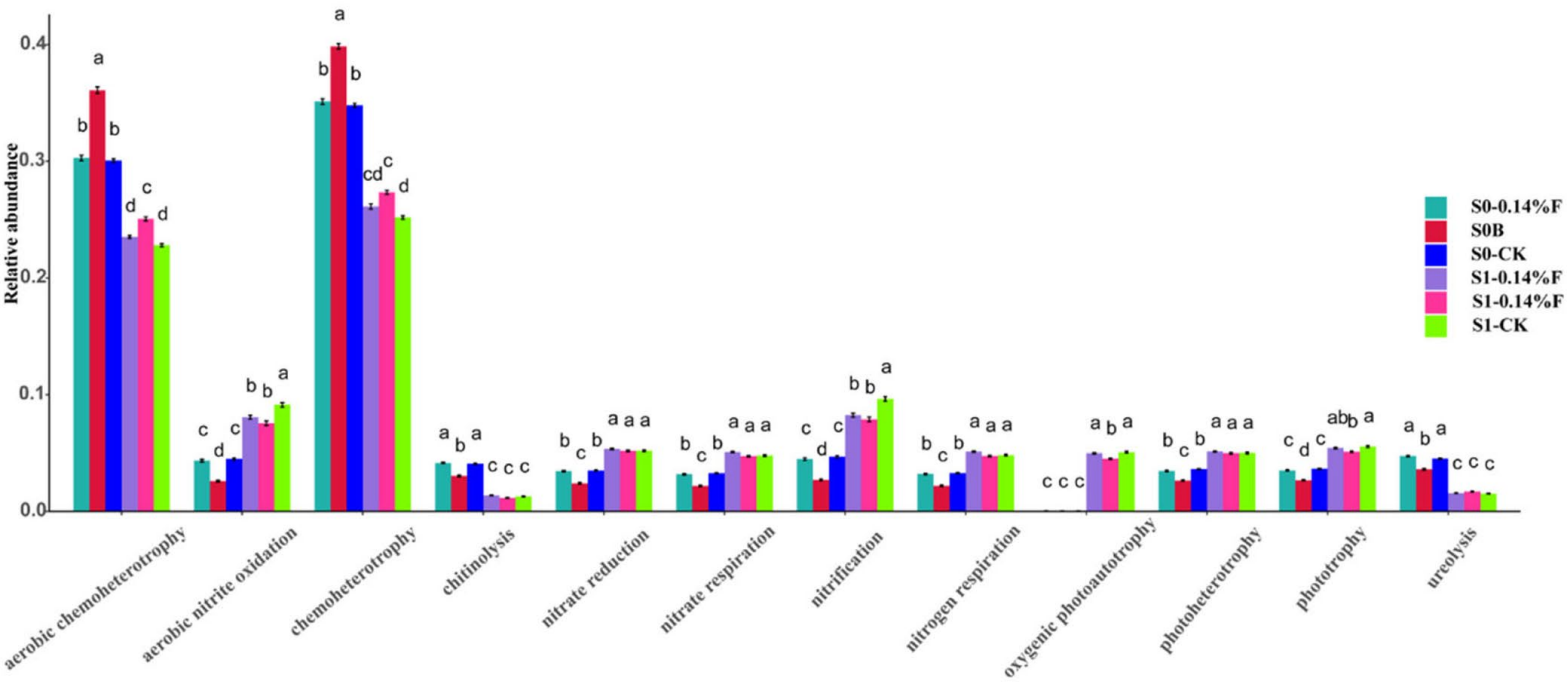
Function predictions of soil microbial communities under different treatments by FAPROTAX. Comparison of dominant functional groups. Different letters indicate significant differences at *p* < 0.05.

At S0 stage, compared with CK, samples from the 0.14%F and 1.4%F groups showed a higher relative abundance of the predicted functional groups for aerobic chemoheterotrophy and chemoheterotrophy. Samples from 1.4%F showed a significantly lower relative abundance of the predicted functional groups for aerobic nitrite oxidation, chitinolysis, nitrate reduction, nitrate respiration, nitrification, nitrogen respiration, and ureolysis (Figure 6). At S1 time point, compared with CK, samples from the 0.14% F and 1.4%F groups showed a higher relative abundance of the predicted functional groups for aerobic chemoheterotrophy and chemoheterotrophy but showed a significantly lower relative abundance of the predicted functional groups for aerobic nitrite oxidation and nitrification.

**FIGURE 7:**
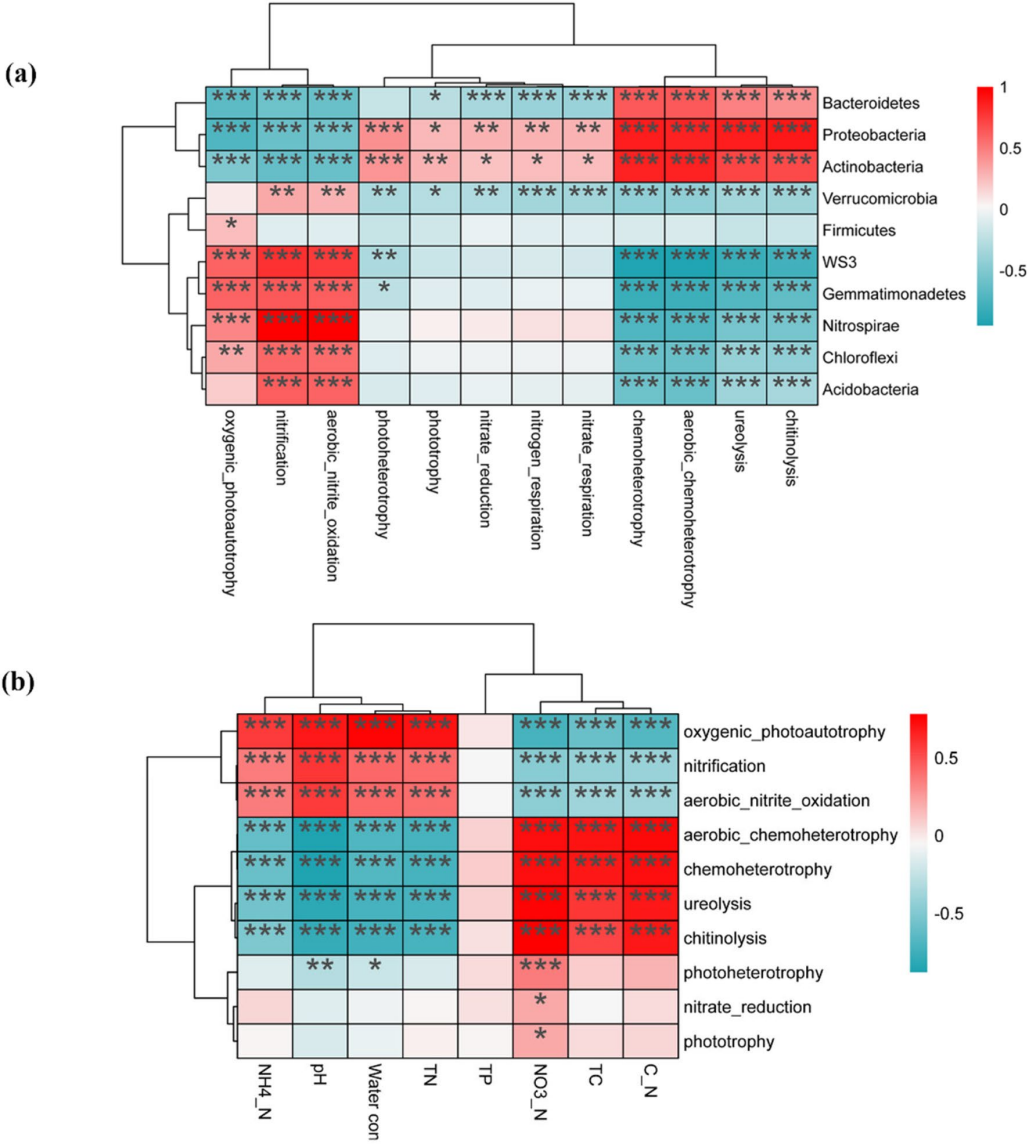
Correlation between microbial classes and predicted functional groups (a). The correlation between predicted functional groups and soil physical and chemical properties (b). Blue and red colors indicate positive and negative correlations, respectively. *p < 0.05, **p < 0.01, and***p < 0.001.

The relationship between soil microbial community and functional group was shown by heat map (Figure 7a). *Bacteroidetes* showed a significantly positive correlation with chitinolysis, ureolysis, aerobic chemoheterotrophy, and chemoheterotrophy but showed a significantly negative correlation with nitrate respiration, nitrogen respiration, nitrate reduction, and phototrophy.

*Proteobacteria* and *Actinobacteria* showed a significantly positive correlation with chitinolysis, ureolysis, aerobic chemoheterotrophy, chemoheterotrophy, nitrate respiration, nitrogen respiration, nitrate reduction, phototrophy, and photoheterotrophy but showed a significantly negative correlation with aerobic nitrite oxidation, nitrification, and oxygenic photoautotrophy.

*Acidobacteria*, *Nitrospirae*, *Chloroflexi*, *Gemmatimonadetes*, and *WS3* showed a positive correlation with aerobic nitrite oxidation, nitrification, and oxygenic photoautotrophy but showed a significantly negative correlation with chitinolysis, ureolysis, aerobic chemoheterotrophy, and chemoheterotrophy.

The relationship of functional groups and soil physicochemical properties was shown by Pearson correlation (Figure 7b). C/N, TC, and NO_3_^−^‐N were significantly positively correlated with aerobic chemoheterotrophy, chemoheterotrophy, ureolysis, and chitinolysis but were significantly negatively correlated with oxygenic photoautotrophy, nitrification and aerobic nitrite oxidation. TN, water content, pH and NH_4_^+^‐N were significantly positively correlated with oxygenic photoautotrophy, nitrification, and aerobic nitrite oxidation, but were significantly negatively correlated with aerobic chemoheterotrophy, chemoheterotrophy, ureolysis, and chitinolysis. The results of the RDA indicated that pH and NO_3_^−^_N were the most important indicators of the structural variation of functional groups (Table 4).

**TABLE 4:** Difference of bacterial community and function explained by soil physicochemical properties by RDA in three treatments.

	Bacterial community structure				Predicted functional groups			
	Explains%	Contribution %	*F*	*p*	Explains%	Contribution %	*F*	*p*
pH	44.4	89.5	70.1	0.002	79	97.4	332	0.002
${\mathrm{NO}}_{3}^{-}$‐N	1.3	2.7	2.1	0.108	1.2	1.5	5.5	0.028
TC	1	1.9	1.5	0.206	0.6	0.7	2.7	0.092
TN	1.1	2.3	1.9	0.142	< 0.1	< 0.1	0.3	0.648
C/N	0.8	1.7	1.3	0.254	< 0.1	0.1	0.4	0.576
NH_4_^+^‐N	0.4	0.8	0.6	0.548	< 0.1	< 0.1	0.2	0.648
Water content	0.4	0.7	0.6	0.608	< 0.1	< 0.1	0.1	0.786
TP	0.2	0.4	0.3	0.816	< 0.1	< 0.1	< 0.1	Unknown

*Note:* Explains % represents the percentage variance in genus level that can be explained by a certain variable. Contribution % represented the percentage of variation that a certain variable contributes to the bacterial community or predicted functional groups. *p* < 0.05 is considered significant.

## 4 Discussion

### 4.1 Effects of Animal Feces on the Soil Physicochemical Properties

The addition of sambar feces significantly affected soil physical and chemical properties. In our study, soil pH significantly decreased after the addition of sambar feces; this finding is similar to the study by Bashir et al. 2021, which may be explained by the following two reasons. (1) Because the sambar feces contain many living microorganisms, decomposition may take place and produce many organic acids after the feces are applied to the soil, thus can reduce the pH of the soil environment. (2) Feces contain a large portion of humus, which is mainly negatively charged and can adsorb a large amount of Ca^2+^ and Mg^2+^ into the soil. The combination of residual H^+^ and SO_4_^+^ ions leads to soil acidification (Lv 1997).

The 0.14% feces concentration did not cause significant changes in soil TC at day 0, but a significant increase in soil TC was observed with the addition of 1.4% feces at the same stage (Table 2) because the carbon content of feces is higher than that of the soil (Table 1). The landslides caused by earthquakes might have turned the original subsoil into a surface layer, which had a shorter time to accumulate carbon from fallen leaves. As a result, there may be a decrease in the carbon content of the surface soil in the damaged ecosystems (Su et al. 2016). We also found that 30 days after the addition of feces, the TC of soil is higher in soil samples with lower concentration of sambar feces. We had hypothesized that there is an optimal range of concentrations where feces can increase the TC of soil, that is too high or too low of feces concentrations will not achieve the optimal effect of carbon enhancement. This finding suggests that the wild ungulates should be managed carefully during the processes of soil restoration in destroyed ecosystems.

In S1 stage, compared with CK samples, the addition of 0.14% and 1.4% sambar feces both resulted in a significant decrease in soil TN content. This could be relevant to microbial utilization of nitrogen. For example, studies have shown that denitrification by microorganisms depletes usable nitrogen and reduces total nitrogen in the soil (Kandeler et al. 2009; Miller et al. 2009).

On day 30, the C/N ratio gradually increased because the TC content increased while the TN decreased. The increase of C/N ratio was shown to possess certain limiting effects on the activities of soil bacteria (Cui et al. 2022), which can lead to the slowing down of the decomposition and mineralization rate of organic matters and organic nitrogen. This can eventually improve the capacity of soil to fix organic carbon (Zhang, Hull, et al. 2011).

### 4.2 Effects of Animal Feces on the Soil Bacterial Diversity

Microorganisms play an important role in the maintenance of soil health (Bruggen et al. 2015). We found that different fecal proportions significantly affected the soil bacterial community. The main conclusion was that the soil bacterial abundance varied in response to the different proportions of feces added to the soil samples. This indicates that the original ecological balance of the soil was altered after the addition of exogenous nutrients, and the abundance and community structure of soil bacteria change accordingly (Zelenev et al. 2005). Although excessive feces could cause significant changes in soil bacterial structure in a short term, these changes would gradually become less significant with the decomposition of feces (Li et al. 2015; Tian et al. 2015).

Different fecal addition treatments also significantly affected the composition of the bacterial community. From the perspective of microbiota composition, *Proteobacteria*, *Acidobacteria*, *Actinobacteria*, *Bacteroidetes*, and *Nitrospirae* are the dominant bacteria, and this result is similar to some previous studies (Zhou et al. 2015; Chen et al. 2022). Further results showed that the addition of sambar feces increased the relative abundance of *Proteobacteria*, *Actinobacteria*, and *Bacteroidetes*. *Proteobacteria* is the largest phylum of bacteria and contains a variety of metabolically active species, which can dominate the C‐rich environment and enhance the nitrogen‐fixation capacity of soil (Yu et al. 2020; Li, Liu, et al. 2022). Researchers noticed that the relative abundance of *Proteobacteria* was positively correlated with soil carbon content (Fazi et al. 2005; Fierer et al. 2007). Similarly, *Actinobacteria* also plays an important role in the soil nitrogen cycle, mainly by participating in the decomposition of organic matter and symbiotic nitrogen fixation (Piao et al. 2008). *Bacteroidetes* serves as the main mineralizers of Total Organic Carbon (TOC), which could increase soil organic carbon content and provide energy for microbial growth and soil enzyme activity (Li, Xu, et al. 2022). *Bacteroidetes* tends to accumulate in soil with high C availability (Zheng et al. 2016). Furthermore, *Bacteroidetes* facilitates enhanced phosphorus uptake by plants and contributes to the regulation of soil nitrogen cycling (Pan et al. 2023). On the basis of this evidence, we think that sambar feces have the potential to increase the abundance of microbial communities involved in soil carbon and nitrogen fixation and thus accelerate the recovery of soil carbon and nitrogen content in destroyed ecosystems, which can in turn promote plant growth.

*Nitrospira* mainly help to transform the nitrite in the soil into nitrate, playing an important role in the nitrogen cycle (Nan et al. 2016). Our results showed that an appropriate amount of sambar feces could increase the relative abundance of *Nitrospira* in the soil, but an excessive amount of sambar feces significantly reduced the relative abundance of *Nitrospira*. This finding indicates that the sambar feces can enrich bacteria involved in the nitrogen cycle and efficiently promote the biogeochemical cycle in soil.

When more feces were added, the relative abundance of *Acidobacteria* was lower. After the feces gradually decomposed, the abundance of *Acidobacteria* gradually increased. *Acidobacteria* are mostly acidophilic bacteria, which belong to oligotrophic bacteria and are not adapted to the soil environment with high nutrient content (Giguere et al. 2021; Xue et al. 2021). This result indicates that the nutrient content of sambar feces is higher than that of the soil in damaged ecosystems.

Although this study was a short‐term controlled experiment, it empirically demonstrated that wildlife feces have a significant effect on soil physicochemical properties and microbial communities. Over longer timeframes and with more extensive plant involvement, different patterns of variations in the properties and microbes of soil may emerge. More comprehensive studies on the interactions among animals, plants, and soil are required to reveal their complex effects on the soil from damaged ecosystems.

### 4.3 Relationships Between the Bacterial Community and Soil Physicochemical Properties

Soil physicochemical properties affect microbial diversity and community structure (Xue et al. 2018; Philippot et al. 2023). In this study, we found that the most important factor affecting the bacterial community was soil pH (Table 4). The decrease of soil pH caused by the addition of feces was the main factor that altered the diversity of the soil bacterial community, which was consistent with the results from other researchers (Zeng et al. 2020; Song et al. 2021). We also found that the bacterial community was affected by soil ${\mathrm{NO}}_{3}^{-}$‐N, C/N, and TN. Nitrogen is an essential nutrient for microorganisms (Xu et al. 2002), and soil C/N ratio is an important index to measure the balance of soil carbon and nitrogen nutrients. A low C/N ratio can accelerate the decomposition by microorganisms and the rate of nitrogen mineralization (Hgberg et al. 2007). ${\mathrm{NO}}_{3}^{-}$‐N concentration was also proved to be a key factor affecting the composition and diversity of soil bacterial communities (Li et al. 2016; Liu et al. 2018; Chen et al. 2025). Overall, we showed that the soil microbial communities can be altered by the different fertilization treatments, potentially through their effects on soil properties, and some of these changes, especially the soil pH and ${\mathrm{NO}}_{3}^{-}$‐N concentration, were significantly correlated with microbial diversity and community structure.

### 4.4 Effects of Sambar Feces on the Function of Soil Bacteria

The results of functional prediction showed that the sambar feces increased the relative abundance of functional bacteria with aerobic chemoheterotrophy and chemoheterotrophy within the soil bacterial community. Aerobic chemoheterotrophy and chemoheterotrophy are considered to possess broad ecosystem functions (Bay et al. 2021; Costa et al. 2024) and are performed by most microorganisms, such as *Proteobacteria* and *Acidobacteria*. Aerobic chemoheterotrophy is the main means of carbon metabolism in aerobic microbial communities (Gu et al. 2019), and chemotrophic bacteria are generally considered important in the process of organic material recycling (Kämpfer et al. 1993). The effects of 1.4%F on the relative abundance of predicted functional groups suggest that excess fecal addition promoted the functions of aerobic chemoheterotrophy and chemoheterotrophy from the bacterial community but inhibited the functions of aerobic nitrite oxidation, chitinolysis, nitrate reduction, nitrate respiration, nitrification, nitrogen respiration, and ureolysis. This is likely due to a shift in the abundance of some sensitive functional bacteria under high concentrations of feces. For example, some species of *Acidobacteria* and *Nitrospirae* associated with the nitrogen cycle showed a lower relative abundance with 1.4%F treatment. Notably, after 1 month of fecal decomposition, the relative abundance of aerobic chemoheterotrophy and chemoheterotrophy bacteria related to carbon cycling was still higher with the fecal treatment than that in the CK, but the relative abundance of aerobic nitrite oxidation and nitrification bacteria, which are related to nitrogen cycling, was lower. This effect might be due to the changes in bacterial composition caused by fecal addition and the interspecific competition between the new dominant species and the related functional species. Alternatively, it may be caused by the accumulation of TC and the consumption of TN in soil. Changes in soil properties can lead to changes in the living environment of soil bacteria, thereby inhibiting the growth and reproduction of certain functional bacteria.

## 5 Conclusions

The sambar feces reduced the soil pH and increased soil TC. It also altered the network structure and key microorganisms of the soil microbiota, which may improve soil carbon and nitrogen cycling as well as potential functions of soil microbiota in the recovery of damaged ecosystems. Moreover, the sambar feces not only altered the correlations between soil physicochemical properties and microbial communities but also induced variations in soil microbial functions. Our study quantified the effects of wild ungulates' feces on soil physicochemical properties, and highlighted the impact of feces on several aspects of soil microbiota composition, diversity, structure, and function. This study is the first step to reveal the mechanism of animal‐plant‐soil coupling in the restoration process of damaged ecosystems. Further studies should explore the link between wildlife feces and plant growth more comprehensively. Those studies may contribute to our knowledge on soil restoration in damaged ecosystems and provide guidance on the management of wildlife during the restoration processes.

## Author Contributions

**Yang Hong:** conceptualization (equal), data curation (equal), formal analysis (equal), methodology (equal), supervision (equal), validation (equal), visualization (equal), writing – original draft (equal). **Jiao Xiang:** conceptualization (equal), data curation (equal), investigation (equal), methodology (equal), software (equal), supervision (equal), validation (equal), visualization (equal), writing – original draft (equal). **Han Wu:** data curation (supporting), software (supporting), visualization (supporting), writing – review and editing (supporting). **Tingfa Dong:** conceptualization (supporting), writing – review and editing (supporting). **Min Xu:** methodology (supporting), writing – original draft (supporting). **Jinyan Huang:** investigation (supporting), methodology (supporting). **Zhenshan Guo:** investigation (supporting), methodology (supporting), software (supporting), validation (supporting). **Yingchun Tan:** data curation (supporting), investigation (supporting). **Zhuo Tang:** data curation (supporting), investigation (supporting). **Lijie Chen:** visualization (supporting), writing – review and editing (supporting). **Zejun Zhang:** formal analysis (supporting), investigation (supporting), methodology (supporting), writing – original draft (supporting). **Jindong Zhang:** conceptualization (supporting), formal analysis (supporting), funding acquisition (supporting), resources (supporting), writing – review and editing (supporting).

## Conflicts of Interest

The authors declare no conflicts of interest.

## Supporting information


**Appendix S1:** ece372423‐sup‐0001‐AppendixS1.docx.

## Data Availability

The relevant data of this paper can be found in the Supporting Information.
