# Supplementary material for: Effects of Wildlife Feces on Soil Properties and Microbiota in the Recovery Processes of Damaged Natural Ecosystem
Source: Ecol Evol. 2025 Oct 30;15(11):e72423. doi: 10.1002/ece3.72423 (PMC12572735; doi:10.1002/ece3.72423)
Supplement: Supplementary file 1 — Appendix S1: ece372423‐sup‐0001‐AppendixS1.docx. [file ECE3-15-e72423-s001.docx]

SUPPLEMENTARY INFORMATION

Effects of wildlife feces on soil properties and microbiota in the recovery processes of damaged natural ecosystem

Appendix S1: Monitoring sites information

Table S1: Basic information for monitoring earthquake- affected sites

| Number | Elevation | Slope | Slope aspect | Disaster type | Length (m) | Width (m) | Matrix composition (%) | | | Vegetation type |
| --- | --- | --- | --- | --- | --- | --- | --- | --- | --- | --- |
|  |  |  |  |  |  |  | Bare rock | Scree fields | Soil |  |
| 1 | 2405 | 60 | 282 | Landslide | 20 | 15 | 0.01 | 0.00 | 0.81 | Deciduous broad-leaved forest |
| 2 | 2405 | 55 | 353 | Landslide | 25 | 20 | 0.01 | 0.00 | 0.14 | Deciduous broad-leaved forest |
| 3 | 2380 | 40 | 292 | Debris flow | 20 | 10 | 0.00 | 0.10 | 0.00 | Deciduous broad-leaved forest |
| 4 | 2621 | 45 | 273 | Landslide | 60 | 8 | 0.02 | 0.00 | 0.00 | Mixed coniferous and broad-leaved forest |
| 5 | 2634 | 45 | 290 | Landslide | 20 | 5 | 0.00 | 0.00 | 0.00 | Mixed coniferous and broad-leaved forest |
| 6 | 2615 | 40 | 270 | Collapse | 80 | 25 | 0.00 | 0.10 | 0.10 | Mixed coniferous and broad-leaved forest |
| 7 | 2360 |  |  |  |  |  | 0.01 | 0.20 | 0.54 | Deciduous broad-leaved forest |
| 8 | 2578 | 50 | 360 | Debris flow | 1200 | 100 | 0.00 | 0.60 | 0.05 | Subalpine dark coniferous forest |
| 9 | 2469 | 45 | 0 | Landslide | 45 | 15 | 0.04 | 0.01 | 0.50 | Deciduous broad-leaved forest |
| 10 | 2416 | 50 | 345 | Landslide | 60 | 35 | 0.05 | 0.10 | 0.05 | Mixed coniferous and broad-leaved forest |
| 11 | 2405 |  |  | Landslide | 60 | 35 | 0.05 | 0.10 | 0.05 | Mixed coniferous and broad-leaved forest |
| 12 | 2369 | 45 | 345 | Landslide | 45 | 15 | 0.04 | 0.01 | 0.50 | Deciduous broad-leaved forest |
| 13 | 2152 | 25 | 254 | Debris flow | 40 | 30 | 0.00 | 0.05 | 0.05 | Mixed coniferous and broad-leaved forest |
| 14 | 2225 | 50 | 253 | Landslide | 80 | 45 | 0.01 | 0.05 | 0.03 | Mixed coniferous and broad-leaved forest |
| 15 | 2311 | 45 | 232 | Landslide | 40 | 20 | 0.10 | 0.40 | 0.25 | Deciduous broad-leaved forest |
| 16 | 2404 | 60 | 320 | Landslide | 50 | 50 | 0.10 | 0.00 | 0.05 | Mixed coniferous and broad-leaved forest |
| 17 | 2511 | 45 | 253 | Landslide | 55 | 10 | 0.10 | 0.25 | 0.00 | Mixed coniferous and broad-leaved forest |
| 18 | Re-collapse completely destroyed this monitoring site | | | | | |  |  |  |  |
| 19 | 2275 | 45 | 230 | Landslide | 60 | 5 | 0.00 | 0.40 | 0.00 | Deciduous broad-leaved forest |
| 20 | 1889 | 45 | 320 | Landslide | 180 | 60 | 0.00 | 0.02 | 0.00 | Deciduous broad-leaved forest |
| 21 | 1810 | 50 | 260 | Landslide | 40 | 30 | 0.00 | 0.05 | 0.00 | Deciduous broad-leaved forest |
| 22 | 1820 | 50 | 338 | Collapse | 5 | 5 | 0.00 | 0.20 | 0.00 | Deciduous broad-leaved forest |
| 23 | 2400 | 50 | 289 | Landslide | 60 | 60 | 0.02 | 0.02 | 0.06 | Mixed coniferous and broad-leaved forest |
| 24 | 2100 | 50 | 320 | Landslide | 25 | 5 | 0.00 | 0.00 | 0.10 | Deciduous broad-leaved forest |
| 25 | 2166 | 45 | 310 | Landslide | 100 | 40 | 0.05 | 0.07 | 0.04 | Deciduous broad-leaved forest |
| 26 | 2463 | 40 | 360 | Landslide | 80 | 30 | 0.01 | 0.00 | 0.14 | Mixed coniferous and broad-leaved forest |
| 27 | 2442 | 50 | 107 | Landslide | 50 | 35 | 0.02 | 0.08 | 0.10 | Mixed coniferous and broad-leaved forest |
| 28 | 2524 | 45 | 90 | Landslide | 5 | 8 | 0.00 | 0.30 | 0.20 | Mixed coniferous and broad-leaved forest |
| 29 | 2490 | 60 | 110 | Landslide | 60 | 60 | 0.00 | 0.05 | 0.05 | Mixed coniferous and broad-leaved forest |
| 30 | 2670 | 50 | 330 | Landslide | 20 | 5 | 0.05 | 0.00 | 0.05 | Subalpine dark coniferous forest |
| 31 | 2633 | 45 | 85 | Landslide | 40 | 30 | 0.01 | 0.03 | 0.06 | Subalpine dark coniferous forest |
| 32 | 2641 | 60 | 102 | Landslide | 30 | 30 | 0.01 | 0.04 | 0.15 | Mixed coniferous and broad-leaved forest |
| 33 | 2526 | 35 | 83 | Landslide | 12 | 7 | 0.00 | 0.35 | 0.00 | Deciduous broad-leaved forest |
| 34 | 2517 | 55 | 330 | Collapse | 10 | 15 | 0.35 | 0.30 | 0.03 | Mixed coniferous and broad-leaved forest |
| 35 | 2520 | 35 | 325 | Collapse | 25 | 15 | 0.08 | 0.00 | 0.00 | Deciduous broad-leaved forest |
| 36 | 2583 | 35 | 144 | Collapse | 5 | 10 | 0.08 | 0.00 | 0.02 | Deciduous broad-leaved forest |
| 37 | 2621 | 45 | 79 | Landslide | 5 | 5 | 0.00 | 0.15 | 0.00 | Mixed coniferous and broad-leaved forest |
| 38 | 2611 | 70 | 90 | Collapse | 60 | 25 | 0.40 | 0.10 | 0.05 | Mixed coniferous and broad-leaved forest |
| 39 | 2583 | 45 | 80 | Landslide | 20 | 15 | 0.03 | 0.02 | 0.05 | Deciduous broad-leaved forest |
| 40 | Re-collapse completely destroyed this monitoring site | | | | | |  |  |  |  |
